# Inhibition of autophagy using 3-methyladenine increases cisplatin-induced apoptosis by increasing endoplasmic reticulum stress in U251 human glioma cells

**DOI:** 10.3892/mmr.2015.3588

**Published:** 2015-04-01

**Authors:** RUIJIAN ZHANG, RUIJUN WANG, QIANXUE CHEN, HONG CHANG

**Affiliations:** 1 Department of Neurosurgery, People’s Hospital of Inner Mongolia Autonomous Region, Hohhot, Inner Mongolia 010017, P.R. China; 2 Department of Radiology, The First Affiliated Hospital of Inner Mongolia Medical University, Hohhot, Inner Mongolia 010050, P.R. China; 3 Department of Neurosurgery, Renmin Hospital, Wuhan University, Wuhan, Hubei 430060, P.R. China; 4 Department of Neurology, People’s Hospital of Inner Mongolia Autonomous Region, Hohhot, Inner Mongolia 010017, P.R. China

**Keywords:** cisplatin, autophagy, endoplasmic reticulum stress, apoptosis, glioma

## Abstract

Cisplatin is one of the most widely used chemotherapeutic drugs; however, the side effects and drug resistance limit its usage. Previous findings have demonstrated that cisplatin kills tumor cells through endoplasmic reticulum (ER) stress, which provides a novel method to minimize cisplatin toxicity and circumvent cisplatin resistance. ER stress induces cell autophagy, cell apoptosis and the complicated regulatory network between them. The role of autophagy in cisplatin chemotherapy remains to be elucidated. 3-Methyladenine (3-MA) is normally used as an inhibitor of autophagy. The present study reveals a significant role of the inhibition of autophagy by treatment with 3-MA and cisplatin in combination in U251 human glioma cells. It was demonstrated that cisplatin induced the ER stress associated with apoptosis and autophagy in U251 cells. Inhibition of autophagy by 3-MA increased the expression levels of protein disulfide isomerase, ubiquitinated proteins, glucose regulated protein 78 and CCAAT-enhancer-binding protein homologous protein, and induced the activation of caspase-4 and caspase-3. Treatment with 3-MA combined with cisplatin increased cisplatin-induced apoptosis by increasing ER stress. Therefore, the inhibition of autophagy has the potential to improve cisplatin chemotherapy.

## Introduction

Cisplatin (*cis*-diamminedichloroplatinum II) is one of the most widely used chemotherapeutic drugs; however, the associated side effects and drug resistance limit its usage (1–3). In order to overcome the problems associated with cisplatin, previous studies have investigated the mechanism by which cisplatin kills tumor cells and the reasons for its side effects and resistance. The mechanism underlying cell apoptosis induction by cisplatin is complicated and remains to be elucidated. It has been demonstrated that cisplatin may have effects on multiple cellular targets in tumor cells, not only on DNA in the nucleus (4–6). Previous studies revealed that treatment with cisplatin induces endoplasmic reticulum stress (ER stress) *in vitro* and *in vivo* (7–9). Cisplatin-induced ER stress-associated apoptosis is hypothesized to be one of the cisplatin-induced pathways, which contributes to its cytotoxicity and is also involved in drug resistance (10). Therefore, targeting ER stress may be a potential strategy to improve the chemotherapeutic effect of cisplatin.

ER stress triggers the unfolded protein response (UPR), which involves the ER molecular chaperone, glucose-regulated protein 78/binding immunoglobulin protein (GRP78/BIP), ER stress sensor protein, protein kinase R-like ER kinase, inositol-requiring enzyme 1 and activating transcription factor 6, and also their downstream signaling pathway. ER stress induces cell autophagy, cell apoptosis and the complicated regulatory network between them, through the UPR system (11). In UPR, autophagy performed a protective role by transporting misfolded proteins for degradation to avoid ER stress-mediated apoptosis (12–14).

The present study analyzed the effect of the autophagy inhibitor, 3-methyladenine (3-MA), on cisplatin cytotoxicity in U251 human glioma cells. The aim of the present study was to clarify the role of autophagy in cisplatin-induced U251 human glioma cell death *in vitro*, and to determine the relationship between ER stress-associated apoptosis and cisplatin-induced autophagy, in order to identify a novel treatment strategy for glioma.

## Materials and methods

### Cell culture

U251 human glioma cells were purchased from the American Type Culture Collection (Rockville, MD, USA) and cultured in Dulbecco’s modified Eagle’s media (Gibco Life Technologies, Gaithersburg, MD, USA), supplemented with 10% (v/v) fetal bovine serum (Gibco Life Technologies) at 37°C with 5% CO_2_.

### MTT assay

Cell viability was determined using an MTT assay. Briefly, the cells (1×10^4^ cells/well) were plated for 24 h in 96-well plates in 200 *µ*l complete medium and exposed to different concentrations of inhibitors for various durations. Each treatment was repeated in six separate wells. The cells were incubated at 37°C with 5% CO_2,_ and MTT reagent (20 *µ*l, 5 mg/ml; Sigma-Aldrich, St. Louis, MO, USA) in phosphate-buffered saline (PBS) was added to each well and incubated for 4 h. The formazan crystals were dissolved in 150 *µ*l dimethyl sulfoxide (Beijing Dingguo Changsheng Biotechnology Co., Ltd., Beijing, China) and the absorbance was recorded at a wavelength of 490 nm using a Microplate Reader (Bio-Tek Instruments, Inc., Winooski, VT, USA). Cell viability was calculated as follows: Cell viability (%) = absorbanc_eexperiment_/absorbance_control_ × 100.

### Western blotting

For protein analysis, the cells were harvested following 12 h treatment, washed with cold PBS and incubated in ice-cold radioimmunoprecipitation buffer, containing 50 mM Tris-HCl (pH 6.8), 0.1% SDS, 150 mM NaCl, 1 mM EDTA, 0.1 mM Na_3_VO_4_, 1 mM NaF, 1% Triton X-100, 1% NP40, 1 mM dithiothreitol, 1 mM PMSF, 1 *µ*g/ml aprotinin, 1 *µ*g/ml leupeptin and 1 *µ*g/ml pepstatin A. The cells were sonicated (Ningbo Scientz Biotechnology Co., Ltd., Ningbo, China) for 30 sec on ice and subsequently lysed at 4°C for 60 min. The cell lysates were centrifuged for 30 min at 12,000 × g and the protein concentration in the supernatants was determined using bicinchoninic acid reagent (Pierce, Rockford, IL, USA). For western blot analysis, lysate proteins (30–60 *µ*g) were resolved on 12–15% SDS-polyacrylamide gel electrophoresis gels and transferred onto nitrocellulose transfer membranes (Whatman, London, UK). The membranes were blocked with 5% non-fat dry milk in buffer, containing 10 mM Tris-HCl (pH 7.6), 100 mM NaCl and 0.1% Tween-20, for 1 h at room temperature and subsequently incubated with the following primary antibodies: Mouse monoclonal anti-PDI antibody (cat. no. sc-166474), mouse monoclonal anti-Grp78 antibody (cat. no. sc-376768), mouse monoclonal anti-CCAAT-enhancer binding protein homologous protein (CHOP) antibody (cat. no. sc-7351), rabbit polyclonal anti-caspase-4 antibody (cat. no. sc-28229), rabbit polyclonal anti-caspase-3 antibody (cat. no. sc-7148), rabbit polyclonal anti-LC3 antibody (cat. no. sc-292354) and mouse monoclonal anti-ubiquitin (Ub) antibody (cat. no. sc-8017) (1:200 dilution; Santa Cruz Biotechnology, Inc., Dallas, TX, USA) overnight at 4°C. The membranes were then incubated with horse radish peroxidase-conjugated goat anti-mouse (cat. no. 31431) or goat anti-rabbit (cat. no. 31466) secondary antibodies (Thermo Fisher Scientific, Waltham, MA, USA) at 1:2,000 dilution for 1 h at room temperature. The immunoreactive bands were visualized by the diaminobenzidine (Sigma-Aldrich) coloration method. The representative bands of proteins were measured with Quantity one v4.62 software (Bio-Rad Laboratories, Inc., Hercules, CA, USA) and analyzed as described previously (15). The protein expression levels were normalized against β-actin and the ratios against β-actin were expressed as the mean ± standard deviation from three independent experiments.

### Immunofluorescence staining and confocal laser microscopy

U251 cells were cultured on coverslips at a density of 5×10^4^ cells/well in 500 *µ*l complete medium and exposed to different concentrations of inhibitors for various durations. Following treatment, U251 cells were washed with cold PBS three times and fixed in 4%(w/v) paraformaldehyde/PBS for 20 min. The cells were washed with cold PBS three times and were digested with protein enzyme K (Beijing Dingguo Changsheng Biotechnology Co., Ltd.) for 1 min and washed with PBS twice. The cells were incubated with 0.1% (v/v) Triton X-100 for 6–10 min, washed once with PBS and subsequently blocked for 30 min in 5% (v/v) non-immune animal serum/PBS. The cells were incubated overnight with the following primary antibodies at 1:200 dilution: Mouse monoclonal anti-protein disulfide isomerase (PDI) antibody (cat. no. sc-166474), rabbit polyclonal LC3 antibody (cat. no. sc-292354) (Santa Cruz Biotechnology, Inc.); and rabbit monoclonal anti-active caspase-3 antibody (cat. no. ab32042; Epitomics, Burlingame, CA, USA), prior to three washes with PBS. The cells were then incubated with the following secondary antibodies at 1:400 dilution: Tetramethylrhodamine (TRITC)-conjugated goat anti-mouse (cat. no. 31660) and fluorescein isothiocyanate/TRITC-conjugated goat anti-rabbit (cat. nos. 31635 and 31670) (Thermo Fisher Scientific) for 30 min in the dark. The cells were washed three times with PBS, treated with Hoechst 33342 (Sigma-Aldrich)/H_2_O (1 *µ*g/ml) for 2 min and washed three times with PBS. The cells were examined on an Olympus FV1000 confocal laser microscope (Olympus, Tokyo, Japan).

### Statistical analysis

All statistical analyses were performed using SPSS 17.0 for Windows (SPSS, Inc., Chicago, IL, USA). Data were analyzed by an one-way ANOVA, and Tukey’s post-hoc test was used to determine the significance for all pairwise comparisons of interest. P<0.05 was considered to indicate a statistically significant difference. The data are representative of three independent experiments performed in triplicate.

## Results

### Cisplatin induces ER stress-associated apoptosis in U251 cells

**Figure 1:**
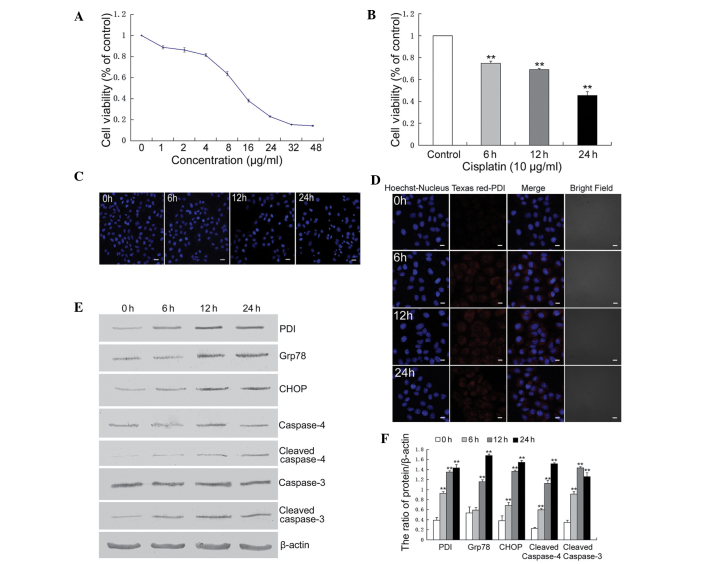
Cisplatin induced endoplasmic reticulum stress-associated apoptosis in U251 cells. (A) U251 cells were treated with varying concentrations of cisplatin for 24 h and cell viability was determined by an MTT assay. (B) The cells were treated with cisplatin (10 *µ*g/ml) for 6, 12 and 24 h, and cell viability was determined by an MTT assay (^**^P<0.01, vs. control). (C) The cells were treated with cisplatin (10 *µ*g/ml) for 6, 12 and 24 h, stained with Hoechst 33342 and cell morphology was observed by confocal microscopy (scale bar, 20 *µ*m). (D) The cells were treated with cisplatin (10 *µ*g/ml) for 6, 12 and 24 h, and the expression of PDI was detected by confocal microscopy (scale bar, 10 *µ*m, Texas red-conjugated secondary antibody). (E) The cells were treated with cisplatin (10 *µ*g/ml) for 6, 12 and 24 h, and western blot analysis was performed to detect the expression levels of PDI, Grp78, CHOP, caspase-4, cleaved caspase-4, caspase-3 and cleaved caspase-3. (F) Quantitation of the protein expression levels. The data are expressed as the mean ± standard deviation (n=3; ^**^P<0.01, vs. Control). PDI, protein disulfide isomerase; Grp, glucose regulated protein; CHOP, CCAAT-enhancer-binding protein homologous protein.

The U251 cells were treated with different concentrations of cisplatin for 24 h or with 10 *µ*g/ml cisplatin for different durations, prior to determining the survival rate using an MTT assay. The results demonstrated that the viability of U251 cells was decreased by treatment with cisplatin in a dose- and time-dependent manner (Fig. 1A and B). The levels of cellular apoptosis in U251 cells treated with cisplatin was assessed by Hoechst 33342 staining. Cisplatin-induced apoptotic chromatin condensation was more evident in the U251 cells following treatment with cisplatin for 12 h compared with the control cells (Fig. 1C). The ER stress associated apoptotic pathway was assessed and treatment with cisplatin upregulated the expression levels of thioredoxin-like PDI, Grp78, CHOP/growth arrest and DNA-damage-inducible protein 153, cleaved caspase-4 and cleaved caspase-3 (Fig. 1D–F).

These results indicated that the ER stress-associated apoptosis pathway was involved in cisplatin-induced U251 cell death.

### 3-MA efficiently inhibits cisplatin-induced autophagy in U251 cells

**Figure 2:**
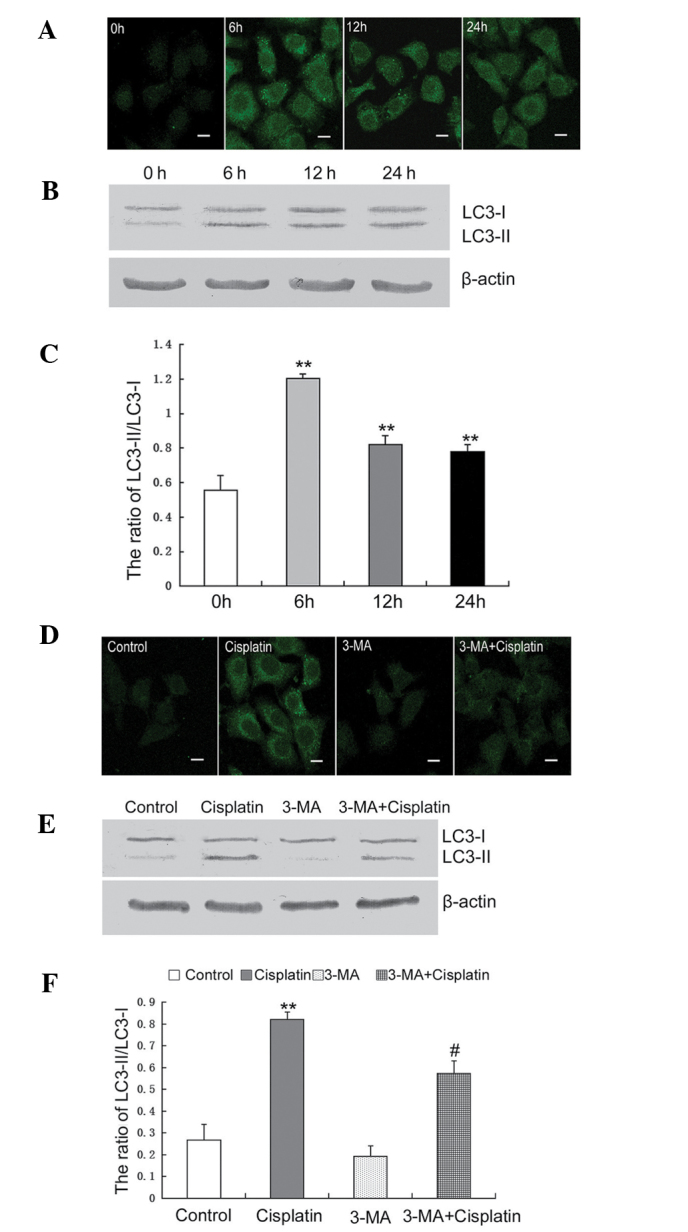
3-MA efficiently inhibits cisplatin-induced autophagy in U251 cells. (A) U251 cells were treated with cisplatin (10 *µ*g/ml) for 6, 12 and 24 h. The expression of LC3 was detected by confocal microscopy (scale bar, 10 *µ*m; FITC-conjugated secondary antibody). (B) The cells were treated with cisplatin (10 *µ*g/ml) for 6, 12 and 24 h, and the expression of LC3 was detected by western blot analysis. (C) The protein levels were quantified for LC3-II/I. The data are expressed as the mean ± standard deviation (n=3; ^**^P<0.01, vs. control). (D) The cells were treated with cisplatin (10 *µ*g/ml) and/or 3-MA (10 mM) for 12 h, and the expression of LC3 was detected by confocal microscopy (Bar, 10 *µ*m; FITC-conjugated secondary antibody). (E) The cells were treated with cisplatin (10 *µ*g/ml) and/or 3-MA (10 mM) for 12 h, and the expression of LC3 was detected by western blot analysis. (F) Quantitation of the protein expression of LC3-II/I. The data are expressed as the mean ± standard deviation (n=3; ^**^P<0.01, vs. control; ^#^P<0.05, vs. cisplatin). 3-MA, 3-methyladenine; LC3, microtubule-associated protein 1 light chain 3; FITC, fluorescein isothiocyanate.

Previous studies have suggested that autophagy can be induced by treatment with cisplatin; therefore, the activation of autophagy was detected. LC3 puncta were observed in U251 cells by confocal microscopy and it was demonstrated that the number of puncta increased following treatment with cisplatin (Fig. 2A). LC3 is a molecular marker of autophagy and is associated with the autophagosome membranes following processing. LC3 transformation was assessed by western blotting and revealed that following treatment with cisplatin, the ratio of LC3II/I was increased at 6, 12 and 24 h (Fig. 2B and C).

Based on these results, the autophagy-specific inhibitor, 3-MA, was used to assess the onset of autophagy in U251 cells treated with cisplatin. Using confocal microscopy, LC3 puncta were observed in U251 cells treated with cisplatin alone and in combination with 3-MA. Following treatment for 12 h, LC3 puncta were clearly observed in cells treated with cisplatin alone and less LC3 puncta were observed in cells treated with cisplatin combined with 3-MA (Fig. 2D). The LC3 transformation in cells treated with cisplatin combined with 3-MA was significantly decreased, compared with the cells treated with cisplatin alone (Fig. 2E and F).

These results indicated that treatment with cisplatin induced autophagy in the U251 cells and this was efficiently inhibited by treatment with 3-MA.

### 3-MA increases cisplatin-induced ER stress

Previous studies demonstrated that cisplatin treatment induces the ER stress response, which regulates autophagy and apoptosis (16,17). Activation of autophagy can attenuate ER stress, therefore, the present study assessed the effect of 3-MA on cisplatin-induced ER stress in U251 cells.

**Figure 3:**
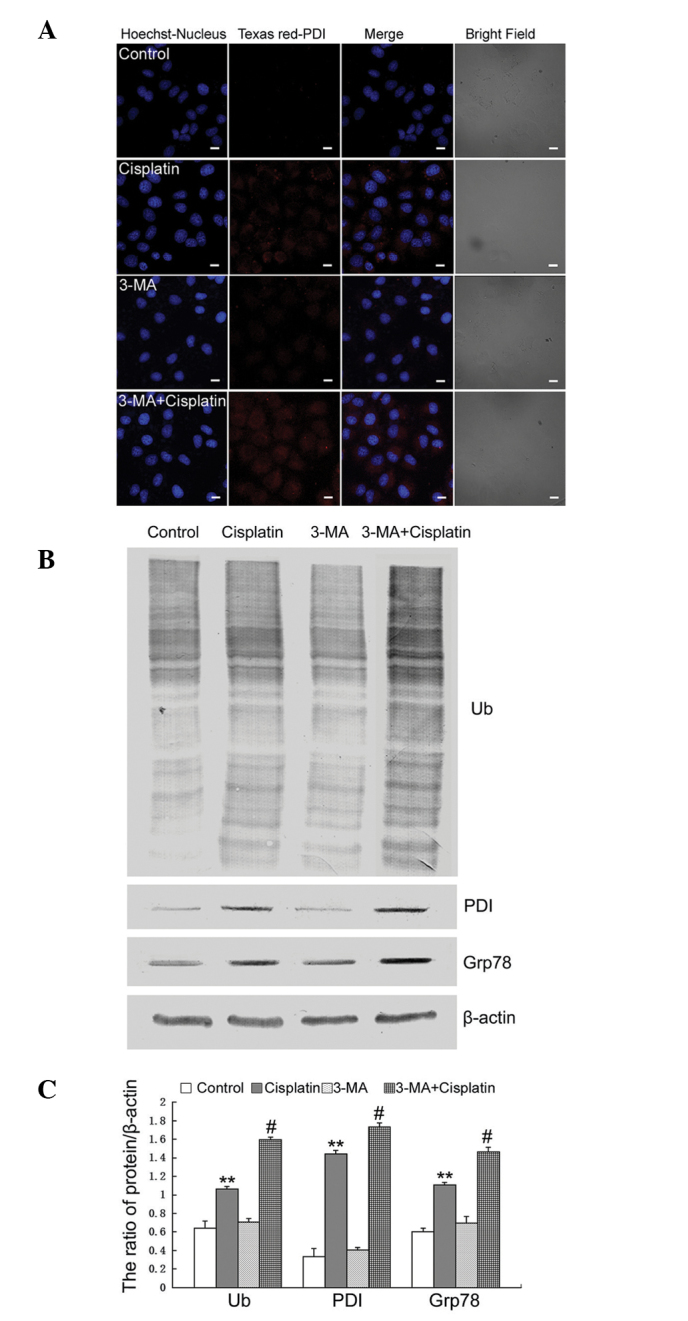
3-MA increases cisplatin-induced endoplasmic reticulum stress. (A) U251 cells were treated with cisplatin (10 *µ*g/ml) and/or 3-MA (10 mM) for 12 h, and the expression of PDI was detected by confocal microscopy (scale bar, 10 *µ*m; Texas red-conjugated secondary antibody). (B) The cells were treated with cisplatin (10 *µ*g/ml) and/or 3-MA (10 mM) for 12 h, and western blot analysis was performed to determine the expression levels of ubquitinated proteins, PDI, and Grp78. (C) Quantitation of the proteins level. The data are expressed as the mean ± standard deviation (n=3; ^**^P<0.01, vs. control; ^#^P<0.05, vs. cisplatin). 3-MA, 3-methyladenine; Ub, ubiqutin; PDI, protein disulfide isomerase; Grp, glucose regulated protein.

The expression of PDI was determined by confocal microscopy. The expression of PDI in cells treated with cisplatin combined with 3-MA was markedly increased, compared with the cells treated with cisplatin alone (Fig. 3A). Western blotting was performed to determine the expression levels of ubiquitinated proteins, PDI and Grp78. The expression levels of ubiquitinated PDI and Grp78 were increased following the inhibition of autophagy by treatment with 3-MA (Fig. 3B and C).

These results indicated that treatment with 3-MA increased cisplatin-induced ER stress by inhibiting autophagy in U251 cells.

### 3-MA increases cisplatin-induced apoptosis by increasing ER stress

Previous studies have suggested that autophagy is important for protecting against cisplatin treatment in tumor cells. Therefore, the present study aimed to detect the effect of inhibiting autophagy on cisplatin-induced apoptosis in U251 cells.

**Figure 4:**
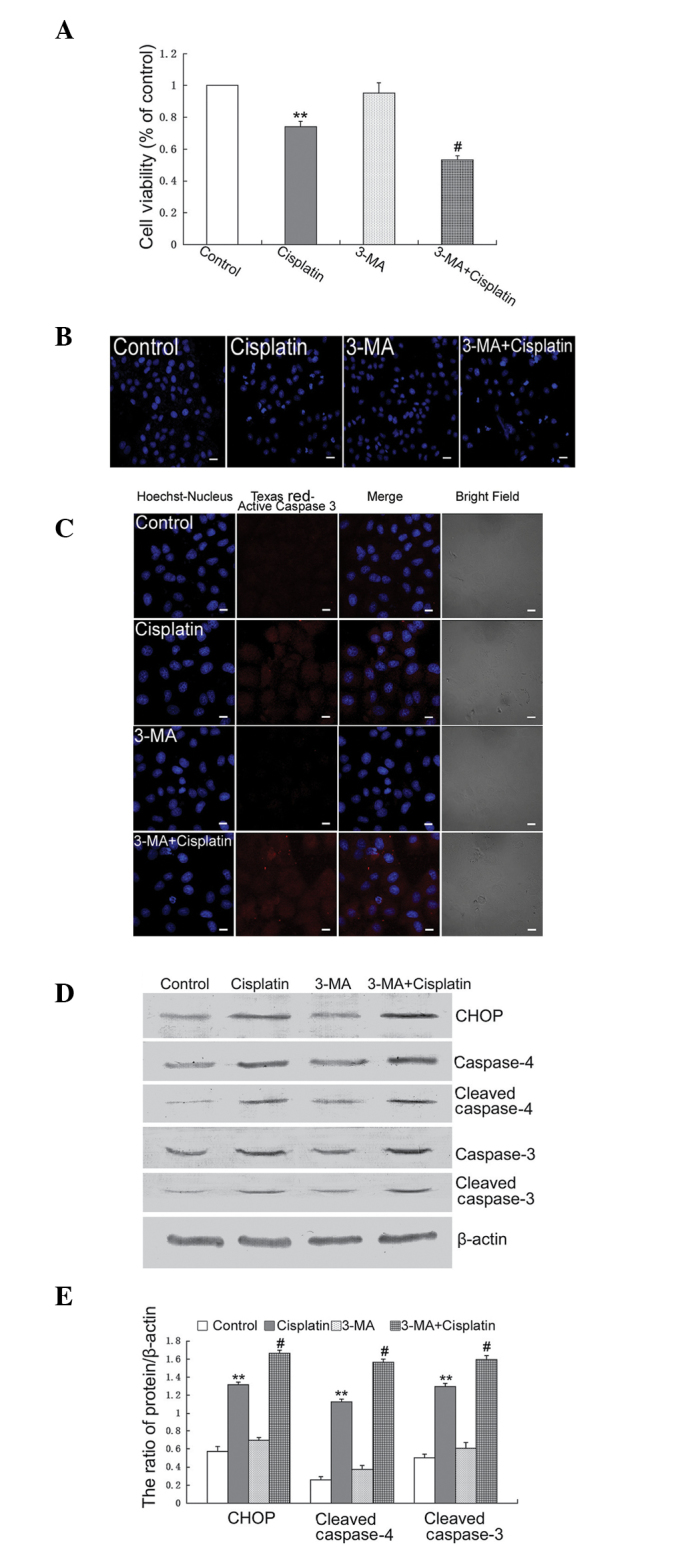
3-MA increases cisplatin-induced apoptosis by increasing endoplasmic reticulum stress. (A) U251 cells were treated with cisplatin (10 *µ*g/ml) and/or 3-MA (10 mM) for 12 h, and cell viability was determined by MTT assay (^**^P<0.01, vs. control; ^#^P<0.05, vs. Cisplatin). (B) The cells were treated with cisplatin (10 *µ*g/ml) and/or 3-MA (10 mM) for 12 h, stained with Hoechst 33342 and cell morphology was observed by confocal microscopy (Scale bar, 20 *µ*m). (C) The cells were treated with cisplatin (10 *µ*g/ml) and/or 3-MA (10 mM) for 12 h, and the expression of cleaved caspase-3 was detected by confocal microscopy (Scale bar, 10 *µ*m; Texas red-conjugated secondary antibody). (D) The cells were treated with cisplatin (10 *µ*g/ml) and/or 3-MA (10 mM) for 12 h, and western blot analysis was performed to determine the expression levels of CHOP, caspase-4, cleaved caspase-4, caspase-3 and cleaved caspase-3. (E) Quantitation of the proteins level. The data are expressed as the mean ± standard deviation (n=3; ^**^P<0.01, vs. control; ^#^P<0.05, vs. cisplatin). 3-MA, 3-methyladenine; CHOP, CCAAT-enhancer-binding protein homologous protein.

It was demonstrated by an MTT assay that treatment with 3-MA increased the growth inhibitory rate in cells treated with cisplatin (Fig. 4A). Using Hoechst 33342 staining, it was demonstrated that 3-MA increased cisplatin-induced apoptotic chromatin condensation (Fig. 4B). Confocal microscopy revealed the expression of active caspase-3. The expression of active caspase-3 was increased in the cells treated with cisplatin combined with 3-MA, compared with the cells treated with cisplatin alone (Fig. 4C). The expression levels of CHOP, cleaved caspase-4 and cleaved caspase-3 were assessed by western blotting and revealed that the expression levels of CHOP, cleaved caspase-4 and cleaved caspase-3 were increased in cells treated with cisplatin combined with 3-MA compared with the cells treated with cisplatin alone (Fig. 4D and E).

These results indicated that treatment with 3-MA increased cisplatin-induced apoptosis by increasing ER stress in U251 cells.

## Discussion

Cisplatin is one of the most efficient chemotherapeutic drugs and is widely used for the treatment of solid tumors, including glioma (18,19). However, side effects and acquired drug resistance limit its application. Although it has a satisfactory effect on several types of tumor, the mechanisms by which is kills tumor cells remain to be elucidated. As a cytotoxic agent, the effect of cisplatin causing tumor cell death is hypothesized to be by DNA damage and the inhibition of DNA synthesis. The DNA damage induced by cisplatin activates multiple signaling pathways, which increase cell death, mainly via the apoptotic pathway (20). Previous studies demonstrated that cisplatin induces ER stress and non-nucleus dependent apoptotic signal activation (7–9).

ER is an important organelle inside the cell and is the location of protein synthesis regulation, protein folding following synthesis and accumulation, stress reaction and calcium ion level modulation. Previous studies indicated that the changes in the microenvironment of tumor cells (glucose deprivation, hypoxia) or antitumor drugs, can induce ER stress, including misfolded and unfolded protein accumulation in the ER and intracellular calcium ion balance abnormality (21–23). ER stress triggers the UPR, which in solid tumors inhibits the majority of translational processes, reduces the protein processing burden in the ER and upregulates molecular chaperones, including Grp78 and Grp94, to increase the ER protein folding capability. Eventually, proteins that fail to be folded correctly will be degraded by the proteasomal and autophagy pathways. When ER stress becomes severe, it induces cell apoptosis by activating the downstream apoptotic signaling pathway (24,25). The decisive factor in this change is CHOP. Increased expression of the transcription factor CHOP, changing the transcription of several proteins, induces the activation of the pro-apoptotic process, activates caspases, integrates mitochondrial events and amplifies the death signal (26,27). The present study demonstrated that cisplatin induced apoptosis in U251 cells in a dose- and time-dependent manner. Treatment with cisplatin increased the expression levels of PDI, Grp78, CHOP, cleaved caspase-4 and cleaved caspase-3, which indicated the activation of ER stress-associated apoptosis.

Autophagy is a reaction of cells to environmental changes. The physical function of autophagy is to degrade macromolecules, including proteins, RNA, redundant glycogen, and aged or damaged organelles in membrane enclosed vesicles, which provides a recycle role to maintain cellular homeostasis (28–30). The formation of the autophagosome is the key event during this process. LC3 exists as two forms, termed LC3-I and -II. LC3-II is associated with the autophagosomal membranes following processing in various cells. The ratio of LC3-II/I is used to assess the levels of autophagy (31). The present study revealed that cisplatin caused the accumulation of LC3 puncta and the transformation of LC3-I to LC3-II, which indicated that cisplatin activated autophagy. Combining the autophagy inhibitor, 3-MA, and treatment with cisplatin revealed that the expression levels of PDI, ubiquitinated proteins and Grp78 were significantly increased. These results indicated that the inhibition of autophagy leads to high level ER stress. Furthermore, increased ER stress increased the expression levels of CHOP, cleaved caspase-4 and cleaved caspase-3, which led to increased cisplatin-induced apoptosis.

In conclusion, the present study demonstrated that cisplatin induced ER stress-associated apoptosis and autophagy in U251 cells. The inhibition of autophagy using 3-MA increased the expression levels of PDI, ubiquitinated proteins, Grp78 and CHOP, and induced the activation of caspase-4 and caspase-3. Treatment with 3-MA combined with cisplatin increased cisplatin-induced apoptosis by increasing ER stress. This indicated that the inhibition of autophagy may be a therapeutic target for the improvement of cisplatin chemotherapy in glioma.
